# Estimation of the relationship between the polymorphisms of selected genes: ACE, AGTR1, TGFβ1 and GNB3 with the occurrence of primary vesicoureteral reflux

**DOI:** 10.1007/s11255-016-1483-9

**Published:** 2016-12-17

**Authors:** Marcin Życzkowski, Joanna Żywiec, Krzysztof Nowakowski, Andrzej Paradysz, Władyslaw Grzeszczak, Janusz Gumprecht

**Affiliations:** 1 Department of Urology, School of Medicine with Division of Dentistry in Zabrze, Medical University of Silesia, Katowice, Poland; 2 Department of Internal Medicine, Diabetology and Nephrology, School of Medicine with the Division of Dentistry in Zabrze, Medical University of Silesia, Katowice, Poland

**Keywords:** Primary vesicoureteral reflux, Genes polymorphism, ACE, AGTR1, TGFbeta1, GNB3, Kidney function

## Abstract

**Purpose:**

Etiopathogenesis of VUR is composite and not fully understood. Many data indicate the importance of genetic predisposition. The aim of this study was to establish the relationship of selected polymorphisms: 14094 polymorphism of the ACE, polymorphism rs1800469 of TGFβ-1, rs5443 gene polymorphism of the GNB3 and receptor gene polymorphism rs5186 type 1 AGTR1 with the occurrence of the primary vesicoureteral reflux.

**Material:**

The study included 190 children: 90 with the primary VUR confirmed with the voiding cystourethrogram and excluded secondary VUR and a control group of 100 children without a history of the diseases of the genitourinary tract.

**Methods:**

The study was planned in the scheme: “tested case versus control.” Genomic DNA was isolated from the leukocytes of peripheral blood samples. The results were statistically analyzed in the Statistica 10 using *χ* ^2^ test and analysis of the variance Anova.

**Results:**

Any of the four studied polymorphisms showed no difference in the distribution of genotypes between patients with primary vesicoureteral reflux and the control group. In patients with VUR and TT genotype polymorphism rs5443 GNB3 gene, the glomerular filtration rate was significantly higher than in patients with genotype CC or CT.

**Conclusions:**

(1) No relationship was found between the studied polymorphisms (14094 ACE gene, rs1800469 gene TGFβ1, GNB3 gene rs5443, rs5186 AGTR1 gene) and the occurrence of primary vesicoureteral reflux. (2) TT genotype polymorphism rs5443 GNB3 gene may be a protective factor for the improved renal function in patients with primary vesicoureteral reflux in patients with genotype CC or CT.

## Introduction

One of the conditions to preserve the homeostasis of the urinary tract is normal outflow of the urine from the kidneys to the bladder. It is a process conditioned physiologically by a specific construction of the distal part of ureter, which, together with the ureteral orifice to the bladder, is a one-way valve to prevent backflow of urine into the kidneys [1–3]. In addition to the anatomy (the importance of the length of submucosal ureter), valve performance also depends on the contractile function of muscles of the bladder triangle and distal parts of the ureter. In the event of failure of the valve mechanism, the backflow of urine from the bladder into the ureter, and then to the kidney occurs [4]. Pathological process of reversing the urine is also conducive to further other factors, among others, increase in intravesical pressure during voiding which comes from neurogenic bladder dysfunction, or the existence of functional or anatomical obstruction in bladder outflow. Clinical experience suggests that vesicoureteral reflux (VUR) is one of the most common diseases of the urinary tract in children [5]. But epidemiological data are inconclusive neither in the subject VUR diagnosis or evaluation of its clinical significance [6–10]. This is due to the fact of physiological remodeling processes of the valve mechanism and changes in bladder function, especially during the first year of life.

This results in a group of sick children, mainly those with a low level of reflux, in a withdrawal of the reflux symptoms and “self-healing” [11]. In the 1960s of the last century observation of Kollermann and Ludwig, during the first 4 years of age, the incidence of asymptomatic VUR decreased in the group spontaneously from 60 to 5% [8, 12]. This was confirmed by a published in 2000 in the Journal of Urology study of the natural history of VUR indicating that in 60–85% of children with the diagnosis made before 30 days of age for an average duration of 20 consecutive months, there has been a spontaneous cure of VUR, in stages III–V, respectively, in 60, 50 and 28% of patients [13]. Therefore, today it is believed that the classical distinction between primary and secondary VUR is too simplistic.

In a situation, where according to the International Reflux Study Committee the “gold standard” for diagnosis of VUR is still voiding cystourethrogram—invasive examination aggravating among others to the exposure to ionizing radiation and urinary tract infection—it is not possible to perform screening in the general population. Thus, the available statistics are mostly the result of analysis of selected population of children with clinical signs or initially suspected for VUR, e.g., on the basis of ultrasound of the urinary tract [9]. In the population of children diagnosed for urinary tract infections, VUR is observed in about 31–59% of the cases [7]. It is estimated that the problem of vesicoureteral reflux applies to 0.4–2% of the total population, and the incidence depends on, among others, age, gender and race [2, 8, 14, 15]. Epidemiological data also suggest 30–50% higher prevalence of this disease in families [10, 16–20].

Backflow of the urine into the kidney may result in the occurrence of recurrent urinary tract infections and progressive damage to the renal parenchyma, which, among others, reinforces in the occurrence of hypertension and the development of reflux nephropathy [5, 8, 21–27]. All of these pathologies can lead to end-stage renal failure and the need to implement renal replacement therapy [14, 17, 28–30].

According to the results of a 10-year follow-up of people under 20 years of age with low glomerular filtration published in 2004 conducted as part of Project ItalKid, vesicoureteral reflux was diagnosed in 25% of patients [31]. According to the database of North America (NAPRTCS Annual Report) from 2003, reflux nephropathy was the fourth cause of end-stage renal failure in children and was diagnosed in 5.3% patients after kidney transplantation and 3.5% of patients on dialysis [14]. The objective quantitative assessment of the problem of progression VUR toward end-stage renal disease in Europe is not possible due to the lack of such data in reports ERA-EDTA and also due to the coexistence of other pathologies of the urinary tract (congenital anomalies of the kidney and urinary tract, CAKUT) [3]. There is the fact that an increased risk of permanent kidney damage, especially at high degrees of vesicoureteral reflux, remains undeniable, and the effectiveness of treatment (conservative or invasive) is not satisfactory [27, 28, 32]. It is sought to factors responsible for the development and progression of the disease, which may in the future be a new cell in diagnosis and/or treatment [33].

In terms of data on the VUR family occurrence, there are high hopes that arouse the results of genetic testing conducted by analysis of candidate genes and more recently—genome association studies (genome-wide association study, GWAS) [34–40]. Research in animal models has shown an association between VUR and mutations of various genes involved in the process of morphogenesis of the urinary tract [1].

## Purpose

The aim of this study was to determine the relationship of selected four polymorphisms with the presence of the primary vesicoureteral reflux:

1. polymorphism 14094 (insertion/deletion) of the angiotensin-converting enzyme gene (angiotensin-converting enzyme, ACE)
2. polymorphism rs5186 receptor type 1 angiotensin II gene (angiotensin II receptor type 1 gene, AGTR1)
3. polymorphism rs1800469 of transforming growth factor beta-1 gene (transforming growth factor beta 1, TGFβ1)
4. rs5443 polymorphism of the beta subunit of the beta-3 protein G gene (guanine nucleotide-binding protein beta subunit G-3 GNB3).

## Materials and methods

The study was planned in the “tested case versus control” scheme. The study was approved by the Bioethics Committee of the Medical University of Silesia in Katowice.

Recruitment for the study group was made through the patients of the Department of Surgery of Children’s Malformations and Traumatology in Zabrze and Ambulatory of Pediatric Urology of the Clinical Hospital nr 1 in Zabrze among children, who are currently, or have been previously diagnosed with primary VUR. The studied group included patients with VUR during bladder filling in the voiding cystourethrogram or passive VUR, when the bladder generates pressure much lower than during micturition, suggesting a failure of valve mechanism. In some cases, the diagnosis was expanded by the urodynamic study (UDS) to exclude functional disorders of the bladder or functional obstruction and the urethrocystoscopy to exclude anatomical obstruction and confirm the incorrect position and width of the ureter orifices.

The exclusion criteria were the finding of secondary vesicoureteral reflux in the course of the subjective and physical examination, urodynamic study and urethrocystoscopy, functional or anatomic obstruction, bladder malfunctions and diagnosis of chronic constipation. The control group consisted of children treated in Department of Surgery of Children’s Malformations and Traumatology in Zabrze for reasons other than the urogenital diseases. The informed consent was necessary to participate in the study made by the patient over the age of 16 years and/or in the case of a minor (under 18 years of age)—his legal guardian.

The study protocol provided a one-time 4 ml venous blood achievement (in order to perform genetic testing) during the child’s stay in the hospital, or his routine visits to the ambulatory. Demographic data (age, height, weight) and information on the age at the diagnosis and current renal function were obtained from medical records of patients. For each patient, based on serum creatinine, glomerular filtration rate (eGFR) was calculated using, respectively, for those under and over 18 years of age—patterns: Schwartz and MDRD (The Modification of Diet in Renal Disease Study equation).

The study involved 190 patients. The study group was comprised of 90 patients with primary vesicoureteral reflux, middle-aged 8.9 ± 4.8 years, including 62% of girls and 38% boys. The average age of the disclosure of the disease was 4.1 ± 2.1 years, and the current serum creatinine level was 0.55 ± 0.18 mg/dl (eGFR 105 ± 17.5 ml/min/1.73 m^2^). The number of patients with diagnosed on the basis of voiding cystourethrogram classified on the basis of reflux grade from I to V was, respectively, 1 (1%) 12 (13%), 42 (47%), 13 (14.5%) and 22 (24.5%). 15% of patients were diagnosed with the coexistence of other diseases of the urogenital system: double pelvicalyceal system (7) and ectopic kidneys (3), cryptorchidism (3).

The control group consisted of 100 children aged 13.5 ± 5.7 years, including 36% of girls and 64% boys.

The isolation of genomic DNA and testing for all of the analyzed polymorphisms were carried out in the Laboratory of Molecular Biology, Department of Internal Diseases, Diabetology and Nephrology in Zabrze, Medical University of Silesia in Katowice.

Each test sample was taken once with 4 ml of venous blood to the S-Monovette Sarstedt Company syringe system, containing potassium edetate. Until the isolation of DNA, blood samples were stored at −200 °C. Genomic DNA was isolated from peripheral blood according to the own modification procedure, using a DNA isolation kit from Epicentre Technologies. The precipitated DNA was dissolved in TE buffer, the concentration was assessed in samples using a NanoDrop spectrophotometer (Thermo Scientific), and working solutions were prepared with a constant DNA concentration of 0.1 mg/ml and 0.01 mg/l.

Genotyping of 14094 (insertion/deletion) in intron 16 of the ACE gene was performed according to published in 1992 protocol Rigat’a et al. [41]. Genomic DNA fragment containing the polymorphic site: insertion/deletion 14094 was grown using PCR (polymerase chain reaction) using the primers: F: 5′-ACC ACT CTG GAG CCC TCT CTT ATC-3′ and R: 5′-GAT GTG GCC ATC ACA GTC TTC AGA-3′. The reaction was done in UNO thermocycler (Biometra) with temperature profile and the visualization and documentation of the results was performed using a gel documentation system (VILBER Lourmat). Length of PCR product evaluated in the standard length of 50 bp DNA Ladder (Fermentas) was 193 base pairs for the D allele (deletion) and 480 base pairs for allele I (insertion).

Genotyping of the remaining three polymorphisms, i.e., polymorphism-509 C/T polymorphism TGFβ rs1800469), C/T at position 825 GNB3 gene (rs5443) and A/C at position 1166 of the gene receptor type-1 angiotensin II receptor (rs5186), was performed with the use of fluorescent probes and TaqMan sets the TaqMan SNP Genotyping Assay (Life Technologies) according to manufacturers instructions.

The results were statistically developed by Statistica 10. Quantitative data are presented as mean ± standard deviation. For the purpose of comparison of distributions of genotypes and alleles of studied polymorphisms, test *χ* ^2^ was used. The analysis of relationship between studied polymorphisms and the degree of reflux vesicoureteral or renal function was made using variance test Anova. Due to the small size of the study group, for the purpose of statistical analysis, patients were divided into 2 subgroups taking into account the clinical course of the disease: a group of VUR (1–2)—patients with low reflux vesicoureteral (i.e., Stage I or II) and a group of VUR (3–5)—patients with a high reflux (i.e., Level III, IV or V).

## Results

### Polymorphism 14094 insertion/deletion gene ACE

The distribution of genotypes in the study group and the control group did not differ significantly and were as follows (respectively, VUR group vs. control group): II 28.9% versus 20.2%, ID 52.2% versus 55.6, 18.9% versus DD 24.2%.

The incidence of allele in VUR group and the control group did not differ significantly and amounted to: allele I and 55% versus 48%, the D allele 45% versus 52%.

There were no differences in the distribution of frequencies of genotypes in the population in relation to the anticipated frequency according to the law Hardy–Weinberg equilibrium.

### Rs5186 polymorphism of the gene AGTR1

There were no statistically significant differences in the distribution of genotypes in the study group and the control group. Specific genotypes were reported in VUR group versus the control group at a frequency of AA versus 61.1% 52.5%, 31.1% versus AC 38.4% CC 7.8% versus 9.1%.

The incidence of allele in VUR group and the control group did not differ significantly and amounted to: allele A 76.7% versus 71.7% C allele (MAF) 23.3% versus 28.3%.

The frequency distribution of genotypes in the population did not differ from anticipated by the law Hardy–Weinberg equilibrium.

### Polymorphism rs1800469 gene TGFβ1

The distribution of genotypes in the study group and the control group did not differ significantly and were as follows (respectively, VUR group vs. control group): CC versus 50% 48.5, 43.3% versus CT 42.4%, 6.7% versus TT 9.1%.

The incidence of allele in the study groups did not differ significantly in VUR group and the control group: C allele 71.7% versus 69.7%, T allele (MAF) 28.3% versus 30.3%.

The frequency distribution of genotypes in the population studied was consistent with the expected according to the Hardy–Weinberg law.

### Rs5443 polymorphism of the gene GNB3

In the VUR group and the control group, the following frequency of genotypes was observed CC versus 55.5% 50.5, 35.5% versus CT 41.4, 9% versus TT 8.1%. There were no statistically significant differences in the distribution.

The incidence of allele in VUR group and the control group did not differ significantly and amounted to: C allele 73.3% versus 71.2%, T allele (MAF) 26.7% versus 28.8%.

The frequency distribution of genotypes in the population studied was consistent with the expected according to the Hardy–Weinberg law.

In the case of any of the investigated four polymorphisms (14094 insertion/deletion gene ACE, gene AGTR1 rs5186, rs1800469 gene TGFβ1 and GNB3 gene rs5443), there was no difference in the distribution of genotypes between patients with vesicoureteral reflux and the control group (Table 1) which indicates the absence of their relationship with the disease.

**Table 1:** Frequency distribution of studied genotype polymorphisms [%]; test *χ* ^2^

Genotype	Polymorphism **14094** ACE gene			Polymorphism **rs5186** AGTR1 gene			Polymorphism **rs5443** GNB3 gene			Polymorphism **rs1800469** TGFβ1 gene		
	II	ID	DD	AA	AC	CC	CC	CT	TT	CC	CT	TT
VUR group	29	52	19	61	31	8	55.5	35.5	9	50	43	7
K group	20	56	24	53	38	9	51	41	8	49	42	9
*p* (VUR vs. K)	ns			ns			ns			ns		

In the case of the ACE gene polymorphisms 14094, TGFβ1 gene rs1800469 and rs5186 gene AGTR1, there were no genotype-dependent differences in glomerular filtration observed. In patients with VUR and the TT genotype of the gene polymorphism rs5443 GNB3, the glomerular filtration rate was significantly greater than in patients with the CC genotype or CT (Tables 2, 3). Comparing subgroups separately by genotype GNB3, no statistically significant differences in age of diagnosis of VUR or observation time were observed.

**Table 2:** Average glomerular filtration depending on the genotype of the polymorphism rs5443 gene GNB3 in the VUR study group

Genotype	eGFR (ml/min/1.73 m^2^)	
	Mean	± SD
CC	103.84	18.0
CT	102.11	12.1
TT	121.70	25.1

**Table 3:** Variance analysis of the polymorphism rs5442 of the gene GNB3 influence on the average glomerular filtration rate

Genotype	*p*
CC versus CT	0.65139
CC versus TT	0.00676
CT versus TT	0.00429

## Discussion

The correct process of developing urinary tract prenatally determines the proper structure and function of the urinary tract, and all disorders lead to developmental defects [3, 42, 43].

The results of researches prove that vesicoureteral refluxes-defects are a group genetically heterogeneous [18, 35, 36, 39, 40]. The defect of the gene may result in different disease phenotypes. This is dependent, among others, on the stage of morphogenesis in which disturbances occurred, as well as environmental factors. Thus, VUR is present not only as an isolated defect, but also as a component for a plurality of congenital syndromes and variable penetration of genes causing the disease often to not reveal clinically and is not recognized [39, 40].

Studies conducted in recent decades show that there are different models of inheritance in predisposition to VUR: autosomal dominant or recessive, sex-linked, or conditioned by multiple genes with variable expression and penetrance of genes. There included the analysis of the relationship of many candidate genes and population studies GWAS [34, 35, 38–40]. Unfortunately, in many cases, the results were inconclusive and even contradictory.

The present study examined the relationship between the occurrence of VUR and selected polymorphisms of four genes involved in the multi-directional homeostasis of urinary tract: two genes encoding components of the renin–angiotensin system (i.e., converting enzyme and receptor type 1 angiotensin II), transforming growth factor beta 1 gene and transmembrane amplifier of biological signals β3 subunit protein G.

### Polymorphism 14094 insertion/deletion gene and ACE gene rs5186 AGTR1

Early embryological studies show the current starting from the 5th week of age, expression of the components of the renin–angiotensin system (RAS) in human fetuses, indicating their important role in the development of urinary tract. The mother and fetus have independent systems RAS. Since the second half of pregnancy fetal RAS undergo the same factors that stimulates mother's RAS, and the concentration of renin and angiotensin II is greater in utero than after birth. Local RAS system takes, among others, role in the regulation of cell division, apoptosis and organogenesis [44]. Mutations in genes encoding components of the renin–angiotensin system lead to the occurrence of developmental disorders of kidney and abnormal regulation of blood volume and blood pressure of the fetus [44]. In adults, the RAS is an important pathogenic factor in the development and progression of many primary and secondary diseases of the urinary tract among others by affecting on the regulation of sodium levels and system pressure, glomerular hemodynamics, and stimulation of mesangial fibrosis [45]. Numerous evidence suggests that the renin–angiotensin system determines multifaceted homeostasis of the body. Interesting reports of Italian researchers connect the polymorphisms of the promoter portion of the gene AGTR1 with longevity [46].

For many years, studies of polymorphic variants of RAS components in terms of their importance in the pathogenesis of many diseases are studied [47, 48].

It has been shown that the DD genotype of the gene polymorphism 14094 angiotensin-converting enzyme (ACE) determines the increased activity of the enzyme, which is essential for the pathogenesis of cardiovascular diseases and renal failure [48]. There is a relationship between the variability and the breed of patients [47–49]. However, research on the relationship of variation of polymorphic insertion/deletion ACE with the occurrence of the primary vesicoureteral reflux or threading kidney disease is inconclusive [50–57]. From published in 2014 meta-analysis, we know that there are no grounds to conclude about the impact of polymorphism for predisposition for VUR, or chronic renal failure [45]. This is confirmed by the results of own research, which showed no significant connection between polymorphic variation (insertion/deletion) of angiotensin-converting enzyme gene in patients with primary vesicoureteral reflux.

Research on A1166C polymorphism (rs5186) gene angiotensin II receptor 1 points to his relationship with an increased risk of coronary heart disease, heart failure and stroke [58]. Known studies are demonstrating the importance of this polymorphism in the incidence of metabolic disorders as metabolic syndrome and the development of hypertension [59, 60].

Angiotensin II vasoconstrictor develops its activity by receptor type 1, affecting in this way the vascular resistance resulting in an increase in blood pressure [45]. Hypertension frequently coexists with kidney disease and is recognized as an independent risk factor of their chronic damage. In the kidneys of patients with vesicoureteral reflux was observed increased expression of type 1 receptor (AT1R) and type 2 (AT2R) angiotensin II [61]. In terms of literature data demonstrating polymorphism rs5186 AGTR1 connection with the occurrence of diabetic nephropathy, we undertook a study to determine whether the above-mentioned polymorphism predisposes to the original vesicoureteral reflux [62]. The results did not show such a relationship. This observation is consistent with the observations of other authors and the results of published in 2014 meta-analysis Braliou et al. [45, 50, 63].

### Polymorphism rs1800469 gene TGFβ1

Transforming growth factor beta (TGFβ1) is a cytokine with a wide spectrum of activities in both the physiological and number of pathological states. On the one hand, it is an important regulator of cell proliferation, growth, differentiation and apoptosis of cells during embryogenesis, and the other plays an important role in tissue repair, activating the immune response to various damaging agents, to stimulate the production and inhibiting the degradation of extracellular matrix proteins and controlling the activity of other cytokines involved in remodeling of tissues [64, 65]. The overexpression of TGFβ1 is a recognized etiological factor in pathological fibrosis of the lungs, liver and kidneys. In many pathologies of the urinary system, including infections of the urinary tract, but also the states of arousal RAAS, there is increased excretion of TGFβ1 [65–67] in urine. There are various, but ambiguous reports concerning the effect of various polymorphisms TGFβ1 to its transcriptional activity or production of the protein [11].

In terms of the wide range of activities, we can expect a multi-pathogenetic effect of TGFβ1 for VUR: both as a morphogenetic causative factor and factor escalating progression toward irreversible damage to the renal parenchyma. Literature of the last decade has brought results of research indicating a link of TGFβ1 polymorphisms both with urinary tract infections, as well as with VUR [11, 38, 66, 67]. However, the impact of studied polymorphisms on the degree of damage to the renal parenchyma remains ambiguous [64, 67, 68]. It is suggested that these polymorphisms are related to damage to the renal parenchyma, not so much in the course of the VUR, but the infection caused by it [30].

However, in our study we found no relationship between polymorphism rs1800469 (formerly described as the T-509C) gene, transforming growth factor beta 1 with the occurrence of primary vesicoureteral reflux.

### Rs5443 polymorphism of the gene GNB3

Part of the cell membrane is composed of three subunits (α, β and γ) G protein (guanine nucleotide-binding protein) and has a GTP-ase activity, which catalyzes the hydrolysis of GTP to GDP. Their role is to transmembrane transfer signals of multiple receptors, including hormones, vasoactive agents, neurotransmitters, and growth factors into intracellular effectors such as adenylate cyclase system, the route of inositol triphosphate or diacylglycerol and ion channels of potassium and calcium [69–75]. This remarkably broad spectrum of activity causes that G proteins are a very important element in the proper functioning of many systems and maintain homeostasis [76, 77]. In 1994, Alfred Gilman and Martin Rodbell received the Nobel Prize for their research on the structure and function of G proteins.

Many of the data show the clinical importance of C825T polymorphism of the gene G protein β3 subunit. Exchange of wild-C allele for allele T (i.e., from cytosine to thymine at position 825 of the gene) results in the occurrence of an alternative way of making the mRNA, resulting in the removal of the protein chain β3 subunit 41 amino acids. Established molecule-Gβ3 is responsible for increasing the biological activity of G proteins [75, 78].

The first studies indicating the role of the C825T polymorphism in the pathophysiology of diseases appeared already in the 1990s of last century, when, among others, Siffert et al. showed a higher incidence of the T allele in patients with hypertension [79]. Further research on this topic, conducted in differing race, among others populations, has brought different, sometimes contradictory results [80–85]. Many authors investigated the relationship between polymorphisms subunit β3 with metabolic abnormalities in terms of carbohydrate and lipid metabolism, obesity, diabetes, hypercholesterolemia and insulin resistance [84, 86–91]. There are also known studies that have been linking a gene polymorphism rs5443 GNB3 with greater risk of cardiovascular disease and Alzheimer’s predisposition or the development and progression of cancer [69, 77, 92–98].

According to available literature data, this is the first study analyzing the connection between gene polymorphism rs5443 GNB3 and the occurrence of primary vesicoureteral reflux.

In our study, we found no statistically significant differences in the distribution of genotypes between people with VUR and the control group, which indicates the absence of a causal link between the mutation of the allele C and the disease. However, it is worth to emphasize that patients with VUR and a homozygous genotype TT of gene polymorphism rs5443 GNB3 have demonstrated significantly greater glomerular filtration rate that the carriers of the C allele so patients with the CC genotype or CT. The interpretation of this finding is ambiguous. Her explanation may be found in the impact of polymorphism GNB3 to modification of glomerular hemodynamics, or the course of interstitial inflammation.

In terms of study by Zeltner et al. [99], it can be concluded that the higher glomerular filtration rate means not improved functions and exponent of more normal nephrons, but is the result of hyperfiltration. In this study, an increased renal blood flow (renal plasma flow) and decreased vascular resistance in not suffering from hypertension carriers of the mutant allele T were shown. Hyperfiltration can be caused by disorders of renal autoregulation causing as a consequence, transferring of high system pressure on the renal glomerulus [100, 101]. Numerous studies indicate the pathogenic importance of carriers of the mutated T allele polymorphism rs5443 in terms of susceptibility to hypertension, having the character of low-renin hypertension [81, 102]. Among the hypothetical mechanisms of its development is taken into account inter alia increased activity of sodium-hydrogen exchanger NHE-1, the function of which 30–50% is mediated by G proteins and increased reactivity of vascular smooth muscle to vasoconstrictive factors, such as acting through G protein, norepinephrine and angiotensin II [81, 103]. Observation of patients after kidney transplantation has brought an interesting suggestion that the higher blood pressure seen in those with the TT genotype polymorphism C825T may be responsible for the deterioration of renal function [104]. In fact, epidemiological studies have shown coexistence of hypertension in approximately 10% of children and 38–50% of adults with vesicoureteral reflux-complicated with renal damage [105]. In the case of own research, we do not have, unfortunately, data on the prevalence of hypertension in patients with VUR, which constitutes a restriction of work and indicates a new direction for further research.

Hyperfiltration is per se considered as a risk factor for the development and progression of renal damage [106–109]. However, there are observations, in which no apparent connection between polymorphism of GNB3 and reduction in GFR, or the occurrence of end-stage renal failure, is found. Studies in patients with biopsy-proven IgA nephropathy showed no effect of carriers of the T allele on the progression of kidney damage, like those conducted among kidney transplant recipients [104, 110]. True Blüthner et al. in a group of dialysis patients with type 2 diabetes observed frequent carriers of the T allele [111]; however, published by Gumprecht et al. [112], analysis of the transfer of the T allele in families whose descendant was treated with renal replacement therapy by dialysis has not proven relationship of polymorphism rs5443 with the occurrence of end-stage renal failure. For a reliable analysis of the hyperfiltration in the group of patients with primary VUR the future studies should include the exact parameters of the kidney filtration and GFR estimated for example by cystatin C levels.

The analysis of literature indicates the second direction of the interpretation of the own results. Well, it can be assumed that the homozygous TT genotype polymorphism rs5443 is a protective factor to achieve a better renal function in patients with VUR. This concept is supported by numerous reports indicating a modifying influence of polymorphism GNB3 on the immune system and inflammation process.

Kidney damage in the course of inflammatory conditions of the urinary system is the result of the virulence of the microorganisms and the reactivity of the immune system and the course of the inflammatory response [113–115]. The boundary between a protective, i.e., directed at a cure, and destructive inflammatory response leading to damage to the kidneys is subtle, and it determines, among others, reactivity of the immune system to invasion of pathogenic factor [114, 116].

Experimental studies in animal models have shown that the barrier epithelium of the urinary tract is controlled by chemokines, including primarily by interleukin-8 (IL-8), and the main effector cells of local defense are neutrophils. Individuals prone to acute interstitial nephritis reveal low CXCR1 chemokine receptor expression [117]. Studies in mice lacking the receptor for IL-8 revealed abnormalities of migration of neutrophils and their accumulation at the epithelium of the urinary tract, resulting in greater damage to the kidney in the course of their interstitial nephritis [118]. Lundsted et al. demonstrated the reduced expression of the receptor for IL-8 in children prone to urinary tract infections.

Histological studies made in rats with acute interstitial nephritis against the infection due to Escherichia coli or Pseudomonas aeruginosa indicate dominance renal interstitial mononuclear cells, mainly of the large T cell morphology and phenotype “helper/inducer.” This points to the role of T cells in the body’s response to invasion of bacteria in the renal parenchyma [119].

Lindemman et al. have shown that carriage of allele T polymorphism rs5443 modulates cellular response and enhances the transformation of lymphocytes after stimulation with antigens. Fraction of CD4+ T cells plays an essential role in antigen presentation, i.e., step necessary to trigger specific immune response [120].

Interleukin-8 develops its effect through the receptor CXC communicating with sensitive to the pertussis toxin (PTX) G protein. In their studies, Virchow et al. showed that carriers of the allele T had enhanced chemotaxis of neutrophils as a result of stimulation of IL-8 and a strong chemoattractant formyl-methionyl-leucyl-phenylalanine (fMLP)—a protein released during bacterial or mitochondrial decay originating from the damaged tissue [110]. The effects of the fMLP are mediated by G protein. Block of G proteins by pertussis toxin disables the effect of fMLP cells [121, 122]. Jahnukainen et al. [114] hypothesized that children with increased response to IL-8 have a pronounced symptoms of infection, leading to early diagnosis, early treatment and the same—to lower renal toxicity. Based on the above data, we consider it more likely that the homozygous genotype TT is a protective factor for longer maintenance of normal renal function in patients with VUR.

## Conclusions

1. No association was found between the studied polymorphisms (14094 ACE gene, the gene AGTR1 rs5186, rs1800469 gene TGFβ1, rs5443 gene GNB3) and the occurrence of the primary reflux vesicoureteral
2. TT genotype homozygous polymorphism rs5443 GNB3 gene may be a protective factor to achieve a better renal function in patients with primary bladder-ureteral reflux.
